# The CD40-CD40L Dyad as Immunotherapeutic Target in Cardiovascular Disease

**DOI:** 10.1007/s12265-020-09994-3

**Published:** 2020-03-28

**Authors:** Laura A. Bosmans, Lena Bosch, Pascal J.H. Kusters, Esther Lutgens, Tom T.P. Seijkens

**Affiliations:** 1 Department of Medical Biochemistry, Amsterdam Cardiovascular Sciences (ACS), Amsterdam University Medical Centers, University of Amsterdam, Meibergdreef 15, 1105 AZ Amsterdam, The Netherlands; 2 Experimental Cardiology, University Medical Center Utrecht, Utrecht, The Netherlands; 3 Department of Pathology, Amsterdam Cardiovascular Sciences (ACS), Amsterdam University Medical Centers, University of Amsterdam, Amsterdam, The Netherlands; 4 Institute for Cardiovascular Prevention (IPEK), Ludwig Maximilian’s University, Munich, Germany; 5 German Centre for Cardiovascular Research (DZHK), partner site Munich Heart Alliance, Munich, Germany

**Keywords:** Cardiovascular disease, Atherosclerosis, Inflammation, Immune checkpoint proteins, CD40, CD40L

## Abstract

Chronic inflammation drives the development of atherosclerosis. Despite optimal treatment of classical cardiovascular risk factors, a substantial portion of the population has elevated inflammatory biomarkers and develops atherosclerosis-related complications, indicating that a residual inflammatory risk drives atherosclerotic cardiovascular disease in these patients. Additional anti-inflammatory therapeutic strategies are therefore required. The co-stimulatory molecule CD40 and its ligand CD40L (CD154) have a central role in the regulation of the inflammatory response during the development of atherosclerosis by modulating the interaction between immune cells and between immune cells and non-immune cells. In this review, we discuss the role of the CD40-CD40L dyad in atherosclerosis, and we discuss recent studies on the therapeutic potential of novel CD40-CD40L targeting strategies in cardiovascular medicine.

## Introduction

Atherosclerosis is a chronic lipid-driven inflammatory disease of the large- and middle-sized arteries and a major underlying cause of cardiovascular diseases (CVD), in particular myocardial infarction (MI) and ischemic stroke [1]. In conjunction with dyslipidaemia, activation of the immune system and subsequent low-grade inflammation plays a critical role in the development of atherosclerosis [1]. Primary and secondary pharmacological prevention by lipid lowering therapies, antiplatelet drugs and antihypertensive agents significantly reduces the incidence of (atherosclerotic) CVD. Despite optimal pharmacological treatment, a substantial part of the population develops atherosclerosis-related complications, indicating that other factors promote atherogenesis in these patients. For example, 30.9% of the patients with stable coronary artery disease and low-density lipoprotein cholesterol levels of < 70 mg/dl have elevated levels of high-sensitivity C-reactive protein (hsCRP), an interleukin (IL)1β- and IL6-induced inflammatory biomarker that is associated with acute cardiovascular events, indicating that a residual inflammatory risk drives atherogenesis in these subjects [1–3].

Several landmark trials, which were published in recent years, provide important insight in the therapeutic potential of anti-inflammatory strategies in atherosclerotic CVD [4–6]. In 2017, the Canakinumab Anti-inflammatory Thrombosis Outcome Study (CANTOS) trial demonstrated that targeted inhibition of the residual inflammatory risk by the anti-IL1β monoclonal antibody canakinumab, reduced recurrent MI and stroke and cardiovascular death (HR 0.85, 95% CI 0.74–0.98) in patients with elevated hsCRP levels (> 2 mg/L) [4]. Despite these beneficial cardiovascular effects, canakinumab is no longer developed for cardiovascular indications, due to an unfavourable cost-effectiveness ratio [4, 7]. In contrast to the CANTOS trial, the Cardiovascular Inflammation Reduction Trial (CIRT), which evaluated the broad-spectrum anti-inflammatory agent methotrexate, failed to reduce MI, stroke or cardiovascular death in high-risk patient populations, e.g. patients with a previous MI or multivessel coronary disease, type II diabetes or a metabolic syndrome [5]. Importantly, median hsCRP levels in this trial were 1.6 mg/L, whereas median hsCRP levels in CANTOS were 4.2 mg/L, suggesting that the median residual inflammatory risk was lower in the CIRT [4, 5]. Also, hsCRP, IL1β or IL6 levels were unaffected by methotrexate [4, 5]. Recently, the Colchicine Cardiovascular Outcomes Trial (COLCOT) investigated the effect of the anti-inflammatory agent colchicine in patients with a recent MI on the composite endpoint of death from cardiovascular causes, resuscitated cardiac arrest, MI, stroke or urgent hospitalization for angina leading to coronary revascularization [6]. After a median follow-up of 22.6 months, colchicine reduced the primary endpoint (HR 0.77, 95% CI 0.61–0.96) [6]. The beneficial effect of colchicine on the primary endpoint resulted from a decreased incidence of stroke (HR 0.26, 95% CI 0.10–0.70) and hospitalization for angina leading to coronary revascularization (HR 0.50, 95% CI 0.31–0.81) [6]. Importantly, no differences in hsCRP were observed between colchicine and placebo treated patients [6]. Together, these landmark trials emphasize that anti-inflammatory therapies have the potential to reduce (recurrent) atherosclerotic CVD, especially if these interventions are targeted at specific atherosclerosis-driving inflammatory pathways in selected patient populations with an elevated residual inflammatory cardiovascular risk, which has fuelled the search for additional immunomodulatory strategies.

One novel strategy to temper inflammation in atherosclerosis is through immune checkpoint proteins, a family consisting of co-stimulatory and co-inhibitory molecules that play a central role in the regulation of inflammation [8]. Immune checkpoint proteins are membrane proteins that are known to regulate T cell activation by providing the second signal after T cell receptor activation. Co-stimulatory molecules boost T cell activation, whereas co-inhibitory molecules hamper cellular activation. In addition to this classical role, immune checkpoint proteins facilitate interactions between various immune cells and non-immune cells, thereby orchestrating the inflammatory response [8]. Given their central role in the regulation of inflammatory processes, modulation of immune checkpoint proteins is a promising anti-inflammatory strategy for atherosclerotic CVD [8, 9]. In this review, we evaluate the role of the immune checkpoint protein CD40 and its ligand, CD40L, in CVD. Furthermore, recent studies on the therapeutic potential of novel CD40-CD40L targeting strategies in cardiovascular medicine are discussed.

### CD40-CD40L: Critical Drivers of (Auto)Immunity

The CD40-CD40L dyad is one of the best characterized immune checkpoint protein pairs and has an important role in the regulation of many immunological processes, including T cell activation, immunoglobulin isotype switching and cytokine production [8, 9]. CD40 is a 43-50 kDa transmembrane protein that belongs to the tumour necrosis factor (TNF) receptor superfamily and is expressed on immune cells, e.g. B cells, dendritic cells (DC), monocytes and macrophages and non-immune cells, including endothelial cells, vascular smooth muscle cells and fibroblasts [8, 10, 11]. Upon interaction with its classical ligand, CD40L (CD154), a 39-kDa transmembrane protein that is mainly expressed on activated T cells and activated platelets, trimerization of CD40 is induced [8, 10, 11]. The relevance of the CD40-CD40L dyad in immunity is emphasized by the clinical phenotype of patients with genetic deficiency of these proteins, causing lethal infections, due to reduced T cell activation and impaired B cell responses [12]. However, genetic deficiency of CD40 or CD40L may also trigger autoimmunity by reducing the numbers of regulatory T cells and increasing autoreactive B and T cells [12]. Given the central role of CD40-CD40L signalling in the regulation of inflammatory processes, this dyad is involved in the pathophysiology of many autoimmune and inflammatory diseases, including inflammatory bowel disease, systemic lupus erythematosus (SLE), rheumatoid arthritis, type 1 diabetes mellitus and allograft rejection, as recently reviewed [13].

### CD40-CD40L Interactions in Atherosclerosis

**Fig. 1:**
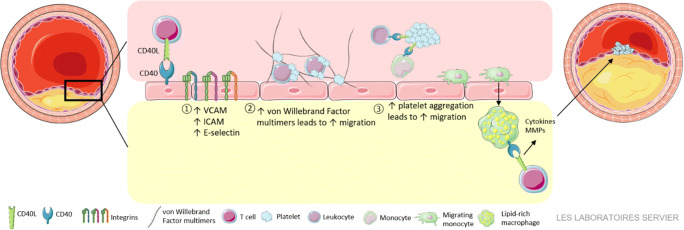
CD40-CD40L interactions in atherosclerosis. CD40-CD40L interactions stimulate leukocyte recruitment to the atherosclerotic plaque by increasing the expression of adhesion molecules on the endothelium [1], inducing the formation of ultra-large vWF multimers [2] and facilitating the formation of platelet-leukocyte aggregates [3]. In advanced atherosclerosis, CD40 activation promotes the production of MMPs, which may contribute to plaque destabilization and rupture

During the development of atherosclerosis, CD40 and CD40L are expressed on the majority of immune cells in the circulation and immune cells and non-immune cells within the atherosclerotic plaque [14, 15]. Extensive research from the last 20 years has unequivocally established the importance of the CD40-CD40L dyad in experimental atherosclerosis. Both genetic deficiency and antibody-mediated inhibition of CD40 or CD40L in mouse models limited the abundance of inflammatory cells within the plaque, reduced atherosclerotic lesion size and enhanced atherosclerotic plaque stability [14–18]. During the formation of initial plaques, CD40-CD40L interactions enhance leukocyte recruitment to the sites of vascular inflammation by at least three mechanisms (Fig. 1). First, CD40L-induced activation of endothelial cells enhances the expression of adhesion molecules, including vascular cell adhesion molecule (VCAM)-1, intercellular cell adhesion molecule (ICAM)-1 and E-selectin [19]. Second, CD40L promotes the formation of net-like ultra-large von Willebrand factor (vWF) multimers on the endothelium, which enhance leukocyte transmigration [20, 21]. Third, platelets CD40L and CD40 facilitate the formation of circulating platelet-leukocyte aggregates, which have an increased potential to migrate into the arterial wall [19, 22]. After migration into the plaque, monocytes differentiate into macrophages and secrete inflammatory effector molecules, such as cytokines and chemokines, which further propagate vascular inflammation. Genetic deficiency or antibody-mediated inhibition of CD40(L) hampers the secretion of these inflammatory mediators, thereby limiting the differentiation of macrophages and T cells towards inflammatory M1 and Th1 subtypes. Absence of CD40 also reduces foam cell formation by limiting CD36 expression on macrophages, which may further limit lesion development [14, 23]. In advanced lesions, CD40-activated macrophages secrete matrix metalloproteinases (MMP), which induce plaque destabilization and rupture [11, 14, 23].

Although these findings identify inhibition of the CD40-CD40L dyad as promising therapeutic strategy in atherosclerosis, antibody-mediated inhibition of CD40 results in severe immune suppression, whereas antibody-mediated blockage of CD40L provokes thromboembolic events due to the disruption of CD40L-αIIbβ3 interactions in arterial thrombi [24]. To exploit the therapeutic potential of CD40 modulating strategies, the downstream signalling pathways that are initiated upon CD40 activation in atherogenesis were explored [25]. CD40 has no intrinsic signalling activity and needs to recruit tumour necrosis factor receptor-associated factors (TRAFs) to initiate intracellular signalling upon activation [14, 25]. The cytoplasmatic tail of CD40 contains a proximal binding site for TRAF6 and a distal binding site for TRAF2/3/5. Deficiency of CD40-TRAF interactions in MHCII^+^ cells in atherosclerotic mice demonstrated that CD40-TRAF6 interactions, and not CD40-TRAF2/3/5 interactions, exert a major role in atherogenesis [25]. Absence of CD40-TRAF6 interactions in MHCII^+^ cells hampered leukocyte recruitment to the plaque, reduced immune cell abundance within the plaque and limited polarization and activation of M1 macrophages, indicating that CD40-TRAF6 interactions predominantly promoted monocyte/macrophage-driven inflammation in atherosclerosis [25]. In contrast, various studies identified a more balanced role for CD40-TRAF2/3/5 interactions vascular biology [25–28]. Deficiency of CD40-TRAF2/3/5 interactions in MHCII^+^ cells of *Apoe*^*-/-*^ mice did not affect atherosclerotic lesion size, plaque inflammation or stability [25]. Accordingly, vascular remodelling and inflammation following carotid ligation was unaffected in *CD40-TRAF2/3/5*^*-/-*^ mice [26]. Genetic deficiency of TRAF2 or TRAF5 reduced the CD40L-induced production of IL6 by endothelial cells, whereas knock-down of TRAF3 in endothelial cells increased the CD40L-induced expression of inflammatory mediators, suggesting a protective role for TRAF3 in atherogenesis [27]. In addition to its role in endothelial cell biology, CD40-TRAF2/3/5 interactions regulate various aspects of B cell activation, such as proliferation and immunoglobulin production, whether deficiency of CD40-TRAF2/3/5 interactions in B cells affects atherosclerosis is currently unknown [29, 30].

Together, these experimental studies highlight a critical role for the CD40L-CD40-TRAF6 axis in monocyte/macrophage-driven inflammation in atherosclerotic CVD, whereas CD40-TRAF2/3/5 interactions have a more balanced role in the regulation of endothelial cell biology. To exploit the therapeutic potential of the CD40-TRAF6 interaction, small molecule inhibitors of this interaction were developed, as described below [31].

### Non-classical CD40 and CD40L Interactions in Atherosclerosis

In addition to CD40, CD40L interacts with at least three other receptors: Mac-1 (αMβ2), VLA-5 (α5β1) and αIIbβ3 [10, 11]. Of these non-classical interactions, the binding of CD40L to the integrin Mac-1 is best characterized in atherogenesis. CD40L-Mac-1 interactions promote monocyte adhesion and recruitment to sites of inflammation [28, 32]. In experimental atherosclerosis models, peptide-mediated disruption of Mac-1-CD40L reduced lesion size, limited macrophage and lesion lipid content and increased collagen content in the aortic root, reflecting a clinically favourable stable plaque, without compromising immunity or thrombosis [32]. CD40L also binds to inactivated VLA-5, which induces the expression of inflammatory mediators, e.g. IL1β, IL6, C-C motif chemokine ligand 2 (CCL2) and MMP2, in monocytic U937 cells, fibroblasts and epithelial cells and has been implicated in the activation of platelets [33–36]. Interactions between CD40L and αIIbβ3 are involved in the stabilization of arterial thrombi, platelet activation and secretion of platelet microparticles [24, 37]. The role of CD40L-αIIbβ3 interactions in atherosclerosis has not been investigated yet, and the therapeutic potential of these interactions may be compromised by its role in thrombus stabilization [24].

Besides CD40L, CD40 has at least two non-classical binding partners: C4b-binding protein (C4BP) and heath-shock protein (HSP) 70 [38, 39]. C4BP directly binds to CD40 on B cells at a site that is distinct from the CD40L binding site and induces B cell proliferation, upregulation of CD54 and CD86 and IgE and IL4 production in an NFkB-dependent manner [39]. Physiologically, this interaction takes place in the germinal centres of secondary lymphoid follicles, such as the tonsil, suggesting that it is involved in the development of B cell–dependent immunity [39]. Although C4BP is present in early and advanced human atherosclerotic lesions, its function is incompletely understood, and it is unknown if C4BP-CD40 interactions occur in the plaque [40, 41]. Microbial HSP70, but not human HSP70, was shown to bind CD40 in myeloid cells, resulting in the production of RANTES, MIP1α and MIP1β [38]. Although HSP70 has been implicated in the pathophysiology of atherosclerosis, it is currently unknown whether this includes interaction with CD40 [42].

Together, these data indicate that non-classical CD40 and CD40L interactions regulate various inflammatory processes during atherogenesis, which may provide novel therapeutic targets to temper inflammation in atherosclerotic CVD.

### CD40 Single-Nucleotide Polymorphisms and Human Atherosclerotic CVD

Genetic studies have identified correlations between single-nucleotide polymorphism (SNP) in the CD40 gene and atherosclerotic risk, in particular the -1T>C SNP in the Kozak consensus sequence of the CD40 gene (rs1883832) [43–47]. A meta-analysis of 7 studies identified a significant correlation between rs1883832 and atherosclerotic CVD in Chinese populations [45]. Recently, Sultan et al. extended these findings to the Caucasian population and also studied the functional consequences of this SNP in endothelial cells [43]. The C allele of the -1T>C SNP of the CD40 gene did not affect CD40 mRNA levels in human umbilical vein endothelial cells (HUVECs) but increased the surface expression of CD40 by 87%, indicating that post-translational regulatory mechanisms were affected by this SNP [43]. These findings are in accordance with a previous study that demonstrated increased expression of CD40 on circulating B cells and monocytes of CC genotype carries compared with TT genotype carriers [46, 47]. Upon soluble CD40L (sCD40L) stimulation of endothelial cells, the CC and CT genotypes expressed higher mRNA levels of TRAF2, whereas TRAF6 expression was unaffected, which resulted in increased expression of E-selectin, VCAM-1 and MCP-1, thereby promoting monocyte adhesion to endothelial cells [43]. Interestingly, the plasma levels of sCD40 were 2.2-fold higher in CC genotype carries as compared with TT genotype carriers with coronary heart disease, which confirmed the clinical relevance of these in vitro findings. It would be interesting to determine correlation between rs1883832 and conventional inflammatory biomarkers, e.g. hsCRP, in CVD patients in future studies.

### sCD40(L): A Biomarker for Atherosclerotic CVD?

In addition to the membrane-bound form, CD40L also exist as circulating molecule, sCD40L, which is mainly derived from activated platelets. Membrane-bound CD40L is cleaved by proteases, e.g. a disintegrin and metalloproteinase domain-containing protein (ADAM)10, ADAM-17, MMP2 and MMP9, to produce circulating sCD40L. sCD40L lacks the cytoplasmatic, transmembrane and part of the extracellular domain [11]. Although its biological effects are incompletely understood, it has been demonstrated that sCD40L promotes platelet activation and induces the expression of matrix metalloproteinase 9 (MMP-9) in endothelial progenitors, which may contribute to plaque destabilization [36, 48, 49].

Many clinical studies have explored the potential of sCD40L as a prognostic biomarker in atherosclerotic CVD [50–54]. For example, in 1088 patients with acute coronary syndrome, elevated serum sCD40L levels were associated with an increased risk for death and recurrent nonfatal MI (HR 2.71; 95% CI 1.51–5.35) during a 6-month follow-up period [51]. In 626 patients with acute chest pain, elevated serum sCD40L levels were associated with detrimental cardiovascular outcomes (HR 6.65; 95% CI 3.18–13.89) [51]. And in 518 patients with ST-segment elevated MI, elevated serum sCD40L levels were associated with a higher incidence of all-cause mortality, cardiovascular mortality and major adverse cardiovascular events, and elevated sCD40L was an independent predictor of 1-year all-cause mortality (OR 3.68; 95% CI 1.54–8.77) [52]. Contrary to these studies, many other studies failed to reproduce these findings [55–61]. Recently, Gergei et al. investigated plasma sCD40L as a biomarker for cardiovascular and all-cause mortality in 2759 persons with a median follow-up of 9.9 years that were included in the Ludwigshafen Risk and Cardiovascular Health (LURIC) study, which is the largest published study on this topic [50]. Plasma levels of sCD40L failed to associate with cardiovascular outcome and all-cause mortality in the whole study population or in various subgroups, such as patients with heart failure, hypertension or coronary artery disease [50].

Several factors may contribute to these heterogeneous results. First, as activated platelets are the main source of sCD40L, the use of antiplatelet drugs, e.g. cyclooxygenase inhibitors or adenosine diphosphate receptor inhibitors, affects the levels of sCD40L [62, 63]. Second, technical issues may affect sCD40L levels, e.g. sCD40L levels are higher in EDTA-plasma compared with citrate plasma, and variations in temperature during plasma isolation affect ex vivo release of sCD40L by platelets [64]. Third, co-morbidities common in patients with atherosclerotic CVD, e.g. hypertension and type 2 diabetes mellitus, increase sCD40L levels, which may hamper the prognostic potential of sCD40L in acute CVD [62, 65]. Finally, a circadian rhythm in sCD40L levels has been observed in patients with acute MI, where sCD40L levels are 41.5% higher in samples obtained at 9 p.m. compared with samples obtained at 2 a.m. [66]. These confounding factors may severely compromise the clinical applicability of sCD40L as biomarker for atherosclerotic CVD.

### The Therapeutic Potential of CD40-CD40L in Atherosclerosis

**Fig. 2:**
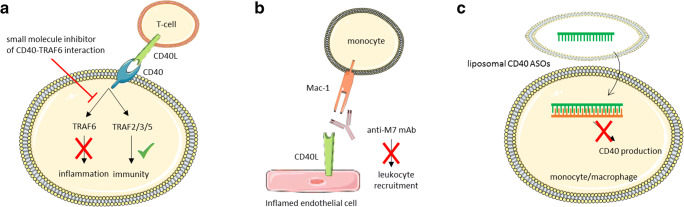
Overview of novel strategies to target CD40(L). **a** Small molecule-mediated inhibition of CD40-TRAF6 interactions prevents inflammation but does not interfere with CD40-TRAF2/3/5-mediated immunity. **b** Antibody-mediated inhibition of the CD40L binding domain of Mac-1 prevents leukocyte recruitment. **c** Liposomal CD40 ASO reduce the expression of CD40 in monocytes and macrophages, but not in B cells

As discussed above, antibody-mediated inhibition of CD40 and CD40L is not clinically feasible due to severe immunosuppression and increased the risk for arterial thromboembolic events [14, 24, 67]. To avoid these limitations, novel strategies were developed to exploit the therapeutic potential of the CD40-CD40L dyad; one example is the development of small molecule inhibitors for the interaction between CD40 and TRAF6, which are named TRAF-STOPs (Fig. 2). These inhibitors, which do not interfere with CD40-TRAF2/3/5 interactions, ameliorated several chronic inflammatory conditions, including insulin resistance and diet-induced obesity by limiting adipose tissue inflammation [31, 68, 69]. In an animal model of non-ischemic heart failure using transverse aortic constriction (TAC), small molecule-mediated inhibition of CD40-TRAF6 reduced adverse cardiac remodelling, improved cardiac function and reduced the influx of macrophages and T cells in the myocardium, which was accompanied by a reduction in cardiac fibrosis and hypertrophy [70]. Small molecule-mediated inhibition of CD40-TRAF6 also hampered the initiation of atherosclerosis in *Apoe*^*-/-*^ mice by reducing inflammatory cell content in the plaques and prevented the progression of established atherosclerosis and induced a stable plaque phenotype [71]. These inhibitors hampered monocyte recruitment to the arterial wall by limiting expression of CD18, CD11a and CD11b on classical monocytes and by reducing CD40-induced NFκB-mediated chemokine and cytokine expression by macrophages [71]. As the CD40-TRAF2/3/5 interactions were not affected, the classical CD40-mediated immune responses, e.g. acute antibacterial responses, DC-mediated co-stimulation, immunoglobulin isotype switching and germinal centre formation, were not impaired [71, 72]. To specifically target myeloid cells, the TRAF-STOPs were incorporated in recombinant high-density lipoprotein nanoparticles [71]. These nanoparticles reduced the initiation of atherosclerosis and were found to be non-toxic in mice and non-human primates, highlighting the therapeutic potential of this strategy in atherosclerosis [71, 72].

Antisense oligonucleotides (ASO) and siRNA have also been explored as a strategy to target CD40. SiRNA-mediated inhibition of CD40 in *Apoe*^*-/-*^ mice reduced atherosclerotic lesions formation, at least partly by limiting CD40-induced NFκB-mediated macrophage activation [73]. However, siRNA-mediated targeting of CD40 also provoked innate immune responses in the kidney, characterized by increased macrophage infiltration and NFκB activation in tubular cells, endothelial cells and infiltrating leukocytes, which may compromise the clinical feasibility of these siRNAs [73, 74]. Alternatively, ASO-mediated targeting of CD40 has been investigated and was shown to reduce inflammation and disease severity in experimental models of colitis and nephritis [75, 76]. By incorporating CD40 ASO in liposomes, macrophages and DCs could more specifically be targeted, without affecting B cells [75]. Although it is currently unknown if this cell type-specific ASO-mediated targeting of CD40 improves atherosclerosis, these data suggest that liposome-mediated delivery of CD40 ASO has the potential to inhibit CD40-driven inflammation in specific immune cell populations without compromising CD40-mediated B cell immune responses.

Non-classical CD40L interactions with Mac-1 have also been explored as therapeutic strategy for inflammatory diseases [32, 77]. As Mac-1 has multiple ligands, e.g. ICAM-1, fibronectin, fibrinogen, RAGE and heparin, and is involved in many biological processes, e.g. immunity and thrombosis, antibody-mediated inhibition is, therefore, not a viable therapeutic strategy in patients [32, 77]. To circumvent the potential problems of general Mac-1 inhibition, Wolf et al. developed a strategy to specifically block the CD40L binding domain (EQLKKSKTL motif) in Mac-1 [32, 77]. The antibody anti-M7 blocks the ligand-binding domain, specifically preventing interaction between Mac-1 and CD40L, but not with other ligands. Anti-M7 reduced the number of adhering, but not rolling, leukocytes to inflamed mesenteric venules of WT mice, whereas this effect was blunted in *Mac-1*^*-/-*^ animals [77]. Accordingly, anti-M7 reduced the number of thioglycolate-induced peritoneal macrophages and did not initiate Mac-1 outside-in signalling to induce inflammatory pathways [77]. Anti-M7 reduced inflammatory cell recruitment, particularly of myeloid cells, in in vivo models of sterile LPS-induced sepsis and did not affect ROS production, suggesting that myeloid effector functions were unaltered. In polymicrobial sepsis, anti-M7 was effective when injected before but not 2 h after the induction of sepsis, highlighting the need for early intervention. Anti-M7 reduced serum amyloid A levels and increased bacterial clearance without affecting vital organs, whereas anti-Mac-1 hampered clearance, potentially by enhancing complement-mediated immune responses driven by iC3b [77]. Thus, specific inhibition of the Mac-1-CD40L interaction improved inflammation in acute models and limited myeloid cell recruitment to the atherosclerotic plaque without compromising host immunity, which highlights the therapeutic potential of this strategy [77].

Although these novel therapeutic strategies did not result in toxicities or immunosuppression in murine models, additional studies in other species will be required before these interventions are translated towards a clinical setting, especially as primary and secondary prevention of atherosclerotic CVD may require long-term treatment. Moreover, a critical selection of patients is required to identify those who will benefit from CD40(L)-targeted interventions. Although hsCRP levels have successfully been used to identify patients with a residual inflammatory risk in the CANTOS trial, novel biomarkers or imaging techniques may be required to select the patients that may benefit from these novel anti-CD40(L) therapies [2].

## Conclusion

Preclinical studies highlight the therapeutic potential of the CD40-CD40L dyad and its non-classical interactions in atherosclerosis (Table 1). Translation of these promising results towards the clinic is challenged by the risk for immunosuppression and thromboembolic events. Novel strategies targeting non-classical CD40L-Mac1 interactions and the inflammatory CD40-TRAF6 interaction overcome the current limitations of CD40(L) targeting interventions and may have the potential to target the residual inflammatory risk that persists after the treatment of conventional cardiovascular risk factors.

**Table 1:** Overview of the role CD40-CD40L and non-classical CD40L interactions in atherosclerosis

Interaction	Cell type	Effect
*CD40-CD40L*	Endothelial cell	• Increased expression of VCAM-1, ICAM-1, E-selectin • Formation of net-like ultra-large vWF multimers • Production of inflammatory mediators, e.g. reactive oxygen species
	Platelet	• Increased formation of circulating platelet-leukocyte aggregates • Platelet activation
	Monocyte	• Increased expression of CD18, CD11a, CD11b on inflammatory monocytes • Enhanced migratory potential of inflammatory monocytes • Increased production of cytokine and chemokine secretion, e.g. TNF, IL1β, IL6, CCL2, CCL5
	Macrophage	• Enhanced migratory potential • Increased production of cytokine and chemokine secretion, e.g. TNF, IL1β, IL6, CCL2, CCL5 • Enhanced foam cell formation • Production of matrix metalloproteinases, plaque destabilization
	Smooth muscle cells	• Increased expression of VCAM-1, ICAM-1, E-selectin • Increased expression of chemokine receptors, e.g. CCR5, CCR1 • IL1β secretion
	Antigen presenting cell	• Enhanced co-stimulatory activity • Production of cytokines, e.g. IL6, IL12, TNF
CD40L-Mac-1	Monocyte	• Increased monocyte adhesion and recruitment to sites of inflammation • Cytokine secretion
CD40L-VLA-5	Monocyte, fibroblast, epithelial cell	• Increased production of cytokines and chemokines, e.g. IL1β, IL6, CCL2 • Production of matrix metalloproteinases
CD40L-αIIbβ3	Platelet	• Stabilization of arterial thrombi • Platelet activation • Secretion of platelet microparticles

## Abbreviations

CVD: Cardiovascular diseases
MI: Myocardial infarction
CRP: C-reactive protein
hsCRP: High-sensitivity CRP
TNF: Tumour necrosis factor
VCAM-1: Vascular cell adhesion molecule 1
ICAM-1: Intercellular cell adhesion molecule 1
vWF: von Willebrand factor
MMP: Matrix metalloproteases
ADAM: A Disintegrin and metalloproteinase domain-containing protein
TRAF: Tumour necrosis factor receptor-associated factors
HUVECs: Human umbilical vein endothelial cells
IL: Interleukin
EAE: Experimental autoimmune encephalomyelitis
SLE: Systemic lupus erythematosus
ASO: Antisense oligonucleotides
